# Editorial: Amoebae as Host Models to Study the Interaction With Pathogens

**DOI:** 10.3389/fcimb.2019.00047

**Published:** 2019-03-19

**Authors:** Sascha Thewes, Thierry Soldati, Ludwig Eichinger

**Affiliations:** ^1^Department of Biology, Chemistry, Pharmacy, Institute for Biology – Microbiology, Freie Universität Berlin, Berlin, Germany; ^2^Department of Biochemistry, Faculty of Science, University of Geneva, Sciences II, Geneva, Switzerland; ^3^Medical Faculty, Center for Biochemistry, University Hospital Cologne, Cologne, Germany

**Keywords:** amoebae, host-pathogen-interaction, evolution, phagocytes, immune system

Amoebae are eukaryotic microorganisms of great diversity. They do not form a single taxonomic group and are found among the protozoa, fungi, and algae. However, all amoebae are characterized by the amoeboid life style—the ability to change cell shape by extending and retracting pseudopods—and most amoebae can be considered as professional phagocytes, which feed on bacteria and other microorganisms by phagocytosis. But despite their impressive phagocytic activity, amoebae cannot degrade all microorganisms. Some resist the digestion and some even use amoebae as host cells for their own replication. Such microbial resistance is also observed in the complex relationship between phagocytes of the mammalian immune system and pathogenic bacteria. For example, *Legionella pneumophila* usually infects fresh-water amoebae, but can also use human alveolar macrophages to further its own replication. This similarity has led to the concept of “amoebae as training grounds for (intracellular) pathogenic bacteria,” namely that pathogenic microorganisms can establish, select and “train” their virulence traits in free-living amoebae before being faced with phagocytic immune cells of animals (Molmeret et al., 2005). Recent studies using amoebae as host models for pathogens confirmed the broad plausibility of this concept. The amoebae used as host models predominantly belong to the phylum Amoebozoa, the closest phylum to fungi and animals, with their most prominent representatives from the genera *Acanthamoeba* and *Dictyostelium*. Both have been demonstrated to be useful host cells for the study of the complex interactions with bacterial pathogens such as *Legionella, Mycobacterium, Salmonella, Francisella*, and others (for reviews see Clarke, 2010; Bozzaro and Eichinger, 2011). Additionally, amoebae can also be used as model hosts for pathogenic fungi like *Cryptococcus, Aspergillus*, and *Candida* (Steenbergen et al., 2003; Chrisman et al., 2011; Hillmann et al., 2015; Mattern et al., 2015; Koller et al., 2016; Maisonneuve et al., 2016). These examples highlight the potential of amoebae as model hosts to study the interaction with a wide range of pathogenic microorganisms. In this Frontiers Research Topic a large range of aspects concerning amoebae as host cells for pathogens is covered.

## Which Amoebae Can be Used as Hosts?

The currently most used and best-investigated amoebae hosts are *Acanthamoeba castellanii* and *Dictyostelium discoideum*. This is highlighted in a review by Swart et al. The authors summarize comprehensively the current knowledge on the interaction of *L. pneumophila* with its natural host *A. castellanii* and the versatile model host *D. discoideum*. The potential of *D. discoideum* as a host model to study different cellular aspects of the interaction with (pathogenic) bacteria is also presented in a method-oriented article by Meena and Kimmel. The authors share a range of protocols using *D. discoideum* to study chemotaxis, phagocytosis, and macropinocytosis.

Further amoebae are used to study specific aspects of the interaction with various pathogens. Diesend et al. summarize the current knowledge about the infection of free-living amoebae with giant viruses. Here, the best-studied amoeba is *Acanthamoeba polyphaga*, which can be infected by a mimivirus. For the study of the interaction of amoebae with pathogenic fungi, the amoebal repertoire is currently the largest. In a review by Novohradská et al. the authors describe the amoebal species that have been used to study fungal interactions. Beside *A. castellanii* and *D. discoideum*, amoebae such as the common water contaminant *Vermamoeba vermiformis, Protostelium mycophagum*, and other mycophagous soil amoebae from the genera *Thecamoeba, Arachnula*, and *Vampyrella* have been used to study the interaction with human-, plant-, and entomopathogenic fungi.

## Which Pathogens can be Studied Using Amoebae?

The range of microorganisms, which can be studied using different amoebae, is not limited to a specific group. Amoebae are already well-established model systems to study the interaction with bacteria. The most prominent bacteria studied using *A. castellanii* and *D. discoideum* are currently from the genera *Legionella* and *Mycobacterium* (see above and reviews by Swart et al.; Cardenal-Muñoz et al.). Two original research articles also highlight this fact. Buracco et al. show that iron depletion or overload of *D. discoideum* cells impacts on the intracellular growth of *Legionella*, whereas changes in zinc and copper concentrations do not have any effect. Concerning mycobacteria, Samba-Louaka et al. show that free-living amoebae might serve as a reservoir for *Mycobacterium avium* subsp. *paratuberculosis*. The authors found that on farms, where cattle were infected with *M. avium* subsp. *paratuberculosis*, the bacteria were also found in environmental samples containing amoebae of the poorly described *Rosculus* genus. *Salmonella enterica* serovar Typhimurium is a further example of bacteria that can be studied using amoebae as hosts. Varas et al. show that inorganic polyphosphate (polyP) is crucial for the virulence of these bacteria. Mutation of the polyP kinase (*ppK*) gene in *S*. Typhimurium affects the ability of intracellular replication. Finally, the spectrum of pathogenic bacteria is constantly expanding. Recently, it has been shown that *Bordetella bronchiseptica* can use amoebae as an environmental niche and transmission vector (Taylor-Mulneix et al., 2017). In a perspective article Taylor-Mulneix et al. now ask whether amoebae are the missing link for the evolution of Bordetellae from environmental microbes to human respiratory pathogens. In this article the concept of amoebae as “training ground” for human pathogens is picked up again. Brock et al. underline this ecological relevance for amoebae as a reservoir for (pathogenic) bacteria in an original research article. The authors isolated environmental samples of *D. discoideum* and found that one third of the wild isolates carried one to six bacterial species per fruiting body. The majority of the isolated bacteria were from the genus Proteobacteria but also Actinobacteria, Bacteriodetes, and Firmicutes have been identified. Many of the bacterial genera isolated in this study include species that are implicated in causing diseases, such as *Brucella, Nocardia*, pathogenic *E. coli, Shigella*, and *Staphylococcus*, suggesting that free-living amoebae can serve as reservoirs for animal and human pathogenic bacteria.

In recent years the use of amoebae as host cells has been expanded to study interactions with different fungi. Novohradská et al. sum up the current knowledge regarding filamentous fungi (human-, plant-, and entomopathogenic). Watkins et al. show in their original research article that the environmental pathogenic yeast *Cryptococcus neoformans* escapes from *D. discoideum* cells after phagocytosis by two different pathways: canonical exocytosis and non-lytic release by vomocytosis, which are the same mechanisms for *C. neoformans* to escape from macrophages.

Last but not least, amoebae as host cells are not restricted to bacteria or fungi. They can also be used to study the interaction with viruses such as the giant mimivirus (Diesend et al.).

All these examples show that different amoebae can be used with a plethora of microorganisms to learn more about the relationship and interaction from an ecological, evolutionary, and medical point of view.

## Which Aspects of Phagocytosis and Killing by the Amoebae are in Common With Mammalian Phagocytes?

Although amoebae are used as host model systems to study interactions with pathogenic microorganisms, one major drawback is that most amoebae grow at environmental temperatures and are usually not able to grow at elevated temperatures such as the human body temperature. However, mammalian phagocytes and amoebae share many conserved cellular and molecular processes concerning phagocytosis and intracellular killing of pathogens. In a mini-review Mori et al. compare and discuss the roles for mammalian and *D. discoideum* coronins in the trafficking and survival of intracellular pathogens. Although many aspects of the regulation and function of coronins are still elusive, a conserved role for coronins in *D. discoideum* and mammals can be delineated. Similarly, mechanisms of host defense processes and intracellular virulence of *Legionella* and *Mycobacterium* are surprisingly highly evolutionarily conserved between animal macrophages and amoebae, as exhaustively reviewed by Swart et al. and Cardenal-Muñoz et al. Additionally, Watkins et al. show that vomocytosis of *Cryptococcus* cells is mechanistically conserved between amoebae and mammalian macrophages. Further, Novohradská et al. nicely compare the parallel events in the phagocytic processing of *Aspergillus* conidiae in mammalian macrophages and amoebae, again supporting the concept of amoebae as training ground for pathogens.

Amoebae are also interesting models to study the early evolution of cell-autonomous, innate immunity (Leippe, 1999), which is reflected in an original research article by Dhakshinamoorthy et al. who investigated the pore-forming saposin-like proteins in the defense against bacterial infection. Such studies show that the limit of amoebae as host models systems is not reached yet.

## Conclusions

It is undisputable that phagocytosis and intracellular processing of pathogenic microorganisms is not identical in every aspect in animal phagocytes and amoebae. However, this research topic and abundant recent publications have illustrated that numerous aspects of these processes are evolutionarily conserved between amoebae and animal phagocytes. Further, the advantages of amoebae as host cells are obvious. Amoebae can be cultivated easily in the lab and—especially in the case of *D. discoideum*—can be easily genetically manipulated. Additionally, many other tools for molecular and cell biology have been established for the use with amoebae.

Such powerful systems will allow to further our understanding of the biology and evolution of host-pathogen interactions. This will constitute the basis for the development of novel anti-infection therapies as it has been shown already for *Mycobacterium* and *Legionella* (Kicka et al., 2014; Harrison et al., 2015; Ouertatani-Sakouhi et al., 2017; Diop et al., 2018; Trofimov et al., 2018). In summary, the future for amoebae as model systems to unravel host-pathogen interactions has just begun.

## Author Contributions

All authors listed have made a substantial, direct and intellectual contribution to the work, and approved it for publication.

### Conflict of Interest Statement

The authors declare that the research was conducted in the absence of any commercial or financial relationships that could be construed as a potential conflict of interest.
